# The Genome of *Fusarium oxysporum* f. sp. *phaseoli* Provides Insight into the Evolution of Genomes and Effectors of *Fusarium oxysporum* Species

**DOI:** 10.3390/ijms24020963

**Published:** 2023-01-04

**Authors:** Yali Hao, Yan Li, Xingxing Ping, Qihong Yang, Zhenchuan Mao, Jianlong Zhao, Xiaofei Lu, Bingyan Xie, Yuhong Yang, Jian Ling

**Affiliations:** 1 Institute of Vegetables and Flowers, Chinese Academy of Agricultural Sciences, Beijing 100081, China; 2 College of Horticulture, Shanxi Agricultural University, Jinzhong 030810, China

**Keywords:** cowpea fusarium wilt, *Fusarium oxysporum* f. sp. *phaseoli*, genomic comparisons, effector

## Abstract

*Fusarium oxysporum* f. sp. *phaseoli*, the causal agent of cowpea fusarium wilt, is a serious threat to cowpea production in China. In this study, a sample of cowpea fusarium wilt was identified as *Fusarium oxysporum* f. sp. *phaseoli* using the methods of morphological characters and molecular detection. We further reported the first genome assembly for *Fusarium oxysporum* f. sp. *phaseoli*, with 53.7 Mb genome sequence comprising 14,694 genes. Comparative genomic analysis among five *Fusarium oxysporum* genomes showed that four accessory chromosomes in the five *Fusarium oxysporum* display similar characteristics, with low sequence similarity (55.35%, vs. overall average of 81.76%), low gene density (2.18 genes/10 kb vs. 3.02 genes/Mb) and highly transposable element density (TEs) (15.01/100 kb vs. 4.89/100 kb), indicating that variable accessory chromosomes are the main source of *Fusarium oxysporum* evolution. We identified a total of 100 *Fusarium oxysporum* f. sp. *phaseoli*-specific effectors in the genome and found 13 specific effector genes located in large insertion or deletion regions, suggesting that insertion or deletion events can cause the emergence of species-specific effectors in *Fusarium oxysporum*. Our genome assembly of *Fusarium oxysporum f. sp. phaseoli* provides a valuable resource for the study of cowpea fusarium wilt, and the comparative genomic study of *Fusarium oxysporum* could contribute to the knowledge of genome and effector-associated pathogenicity evolution in *Fusarium oxysporum* study.

## 1. Introduction

The ascomycete fungus *Fusarium oxysporum* (FO) is one of the soil-inhabiting fungi causing vascular wilt or root rot in over 120 economically important crops worldwide [1]. According to their pathogenicity to a particular host plant, FO can be divided into more than 100 forma specialis (f.sp.), and some forma specialis of FO can be further divided into several physiological races [2]. *Fusarium oxysporum* f. sp. *phaseoli* (FOP), the causal agent of fusarium wilt in many crops in the legume family, is considered as one of the most important soil-borne diseases affecting legume crops [3]. FOP can penetrate plants through the root system and colonizes the xylem, causing wilting, vascular discoloration, chlorosis, dwarfism and premature plant death [4]. Based on the pathogenicity with different common bean genotypes, FOP was reported to have seven physiological races, with different regions having different physiological races around the world [5]. However, with the cultivation of resistant varieties, new virulent groups of FOP have also been detected [6]. Cowpea belongs to the genus *Vigna* of the legume family which includes seven subgenera and more than eighty subspecies [7]. Cowpea is rich in protein, vitamins, calcium, iron and other elements. According to statistics, the area for cowpea cultivation around the world is about 14.5 million hectares [8]. It is planted all over China and is one of the main vegetables in summer and autumn [9]. However, cowpea fusarium wilt (CFW) is one of the important factors affecting cowpea yields, and the annual yield loss is as high as 30–100% around the world [10]. CFW caused by FOP was first reported by Xiao et al. in Hainan province, China, and so far, most FO strains isolated from diseased cowpea plants in China were FOP [8].

The pathogenicity and forma specialis of fusarium species have always been at the forefront of FO research. Based on sequence similarity for the EF-1a gene and ITS sequences, individuals from different forma specialis may be more closely related to each other than individuals in the same forma specialis, suggesting that pathogenicity to certain plant hosts has independently arisen multiple times from distinct lineages [11,12,13]. In addition, to obtain better resolution at the genus or species level in FO, the analysis of multi-locus sequences is necessary, including beta-tubulin, RPB2 (RNA polymerase 2) sequences and so on [14,15], since it adds valuable information in the resolution of complex evolutionary relationships [16]. At present, more than 200 FO genome assemblies are available in the NCBI database, with genome sizes ranging from 44 to 72 Mb [17,18]. FO genomic data greatly accelerate the gene function studies related to pathogenicity and also provide data resources for comparative population genomics, which can better understand the genetic relationships among different FO species [18]. Most FO genomes consist of core chromosomes (CCs) and accessory chromosomes (ACs) [17]. CCs are conserved and vertically transmitted to carry out essential housekeeping functions, while ACs are variable to adapt to new environments or hosts [19,20]. A study of ACs in FO showed that horizontal chromosome transfer of one entire AC can change the host-specific pathogenicity in *F. oxysporum* f. sp. *lycopersici* (FOL) and *F. oxysporum* f. sp. *radices* (FOR) [21].

Pathogens often rely on kinds of small, secreted proteins (effectors) to facilitate the colonization process for successful infection of their host [22]. More and more studies have shown that the pathogenicity of FO is determined by effectors [23,24,25]. Researchers have identified 14 SIX (secreted in xylem) effectors from the xylem sap of tomato plants infected with FO in which some effectors were associated with pathogenicity toward tomatoes [23,26]. Most SIX effectors were related to the pathogenicity of FO, and the pathogenicity of FO decreased dramatically when the SIX genes were knocked down [27]. It was noted that the DNA transposon miniature impala (mimps) TE elements can be detected in the promoter region of these SIX genes and some other virulence-associated genes, by which the candidate effectors can be predicted in different FO genomes [28,29]. FO strains that are pathogenic toward the same host have a similar set of effectors, presumably enabling them to cause disease symptoms in that host [29].

So far, knowledge about the genome information and genetic diversity of FOP is still limited. In this study, we reported the genome assembly of FOP. The FOP genome resource can provide useful help in the study of cowpea fusarium wilt. Our comparative genome analysis among five FO genomes revealed that the accessory chromosomes in FO could play important roles in FO evolution. Effector profiles analysis showed that INDEL events are associated with the emergence of FOP-specific effectors. Our genome resources and comparative genome analysis can deepen our understanding of FO evolution.

## 2. Results

### 2.1. Morphological and Molecular Identification of the Strain Used in This Study

**Figure 1:**
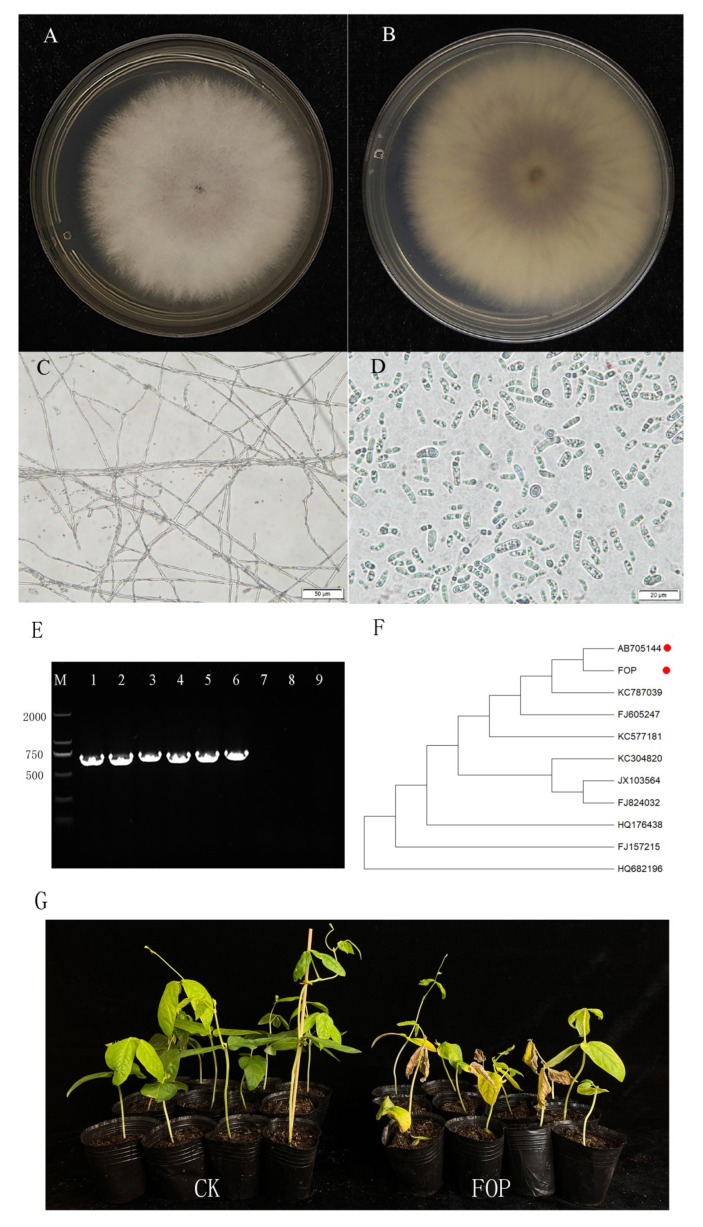
The morphological, molecular identification and pathogenicity test for FOP. (**A**,**B**) Observed colony morphology of FOP from the front (**A**) and back (**B**) of the culture dish. (**C**,**D**) Microscopic observation of mycelia and conidia. (**E**) PCR molecular identification using FO-specific primer. M: marker 2 k. Lane 1–6: the FO with different hosts. Lane 1: FOP. Lane 2: FO host is balsam pear. Lane 3: FO host is eggplant. Lane 4: FO host is bean. Lane 5: FO host is cotton. Lane 6: FO host is watermelon. Lane 7: *Colletotrichum gloeosporioides*. Lane 8: *Botrytis cinerea*. Lane 9: water control. A 729 bp FO-specific DNA fragment can be amplified in all FO strains. (**F**) Phylogenetic tree based on ITS sequences of FOP and another 10 different FOs (only display their accession number in NCBI). The red circle marked AB705144 is the published ITS sequence of FOP. (**G**) The pathogenicity test of FOP. The cowpea seedlings displayed typical fusarium wilt symptoms at 10 days after inoculation with FOP.

The morphological analyses of the strain were performed to check colony morphology, mycelia and conidia characteristics. As shown in Figure 1A,B, colony characteristics of the strain show fluffy growth patterns with white mycelial color. The hyphae are nearly round, the aerial hyphae are white and flocculent, the medium is light purple in the middle and purple on the back, and the mycelium of the strain is dense and concentric whorled. The hyphae are filamentous, colorless and septate (Figure 1C). The microconidia are hyaline, oval-ellipsoid to cylindrical, erect or slightly curved, with average size of 10.82 μm × 3.64 μm (Figure 1D). These characteristics agree with the description of FO [30].

We further performed PCR-based molecular detection by using FO-specific primers. Five other FO forma specialis and two other fungi were selected as control. As shown in Figure 1E, each of the six FOs can be amplified a 729 bp FO-specific DNA fragment, which was not detected in the other two non-FO fungi. As a result, we determined the strain as FO. Furthermore, the ITS sequence of the strain showed the highest identities (99.64%) with published FO strain sequences when blasted against the NCBI database. An ITS-based phylogenetic tree was constructed using ITS sequences of another 10 different FOs. As shown in Figure 1F, the ITS sequence of the strain was clustered with a published FOP sequence (accession number: AB705144), indicating that they have the closest genetic distance. The pathogenicity test of the strain showed that after 10 days, the inoculated seedlings displayed typical fusarium wilt symptoms, including wilting, yellowing leaves and plant dwarfing, whereas the control remained unaffected (Figure 1G and Figure S1). Based on the above results, we confirmed the strain used in this study as FOP.

### 2.2. Phylogenetic Evolution Analysis of FOP

**Figure 2:**
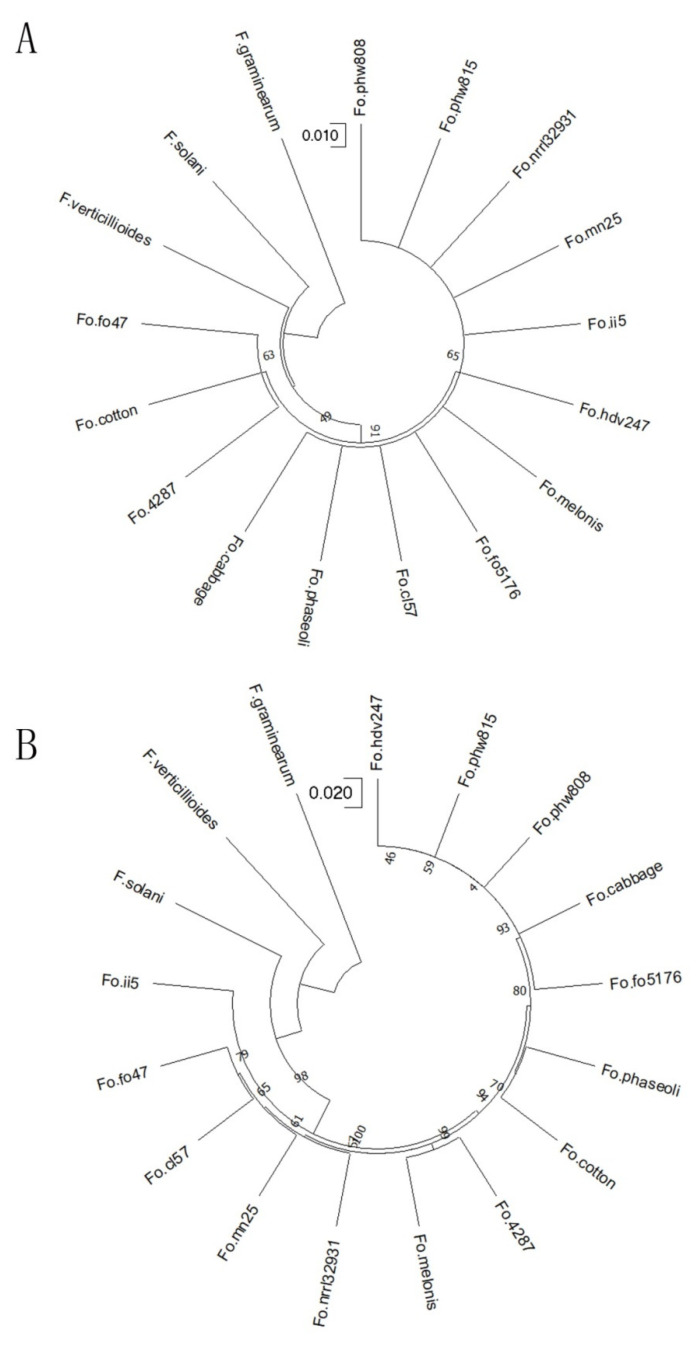
Phylogenetic tree constructed by ITS (**A**) and EF-1a (**B**) gene sequences using the neighbor-joining (NJ) method with bootstrap values shown at nodes. Fo.nrrl32931 is an *F. oxysporum* strain which can infect humans. Fo.cl57, Fo.mn25 and Fo.4287 are different strains of FOL (*F. oxysporum* f. sp. *lycopersici*). Fo.phw808, Fo.cabbage and Fo5176 are different strains of *F. oxysporum* f. sp. *conglutinans*. Fo.fo47 is an *F. oxysporum* biocontrol strain isolated from soil. Fo.cotton: *F. oxysporum* f. sp. *vasinfectum*. Fo.melonis: *F. oxysporum* f. sp. *melonis*. Fo.hdv247: *F. oxysporum* f. sp. *pisi*. Fo.ii5: *F. oxysporum* f. sp. *cubense*. *Fo*. *phaseoli* is the FOP used in this study.

To analyze the evolutionary relationship of FOP in FO species, the ITS, EF-1a, beta-tubulin, RPB2 (RNA polymerase 2) and CYP51C sequences of FOP and 16 other fusarium species (including 13 FO species) were used to construct phylogenetic trees, and *F. graminearum* (FG) was used as an outgroup. All phylogenetic trees showed that 14 FO species can be distinguished from other fusarium species (Fg, *F. verticillioides* (FV) and *F. solani* (FS)) (Figure 2 and Figure S2). The sequence similarity analysis also supports the result. For EF-1a sequences, the average sequence similarity value among FO species is 98.36%, while FG, FV and Fs share an average of 87.14% similarity with FO species. For ITS sequences, the values are 99.64% and 93.23%, indicating that there are significant differences among different fusarium genera. It was noted that the sequence similarity among FO species of EF-1a sequences is lower than that of ITS sequences, suggesting that EF-1a sequences may be more able to display the variation among FO species. Compared with the ITS sequence, EF-1a, beta-tubulin, RPB2 and CYP51C sequences can better reflect the phylogenetic relationship among *Fusarium* genera. For example, EF-1a, beta-tubulin, RPB2 and CYP51C phylogenetic tree support FS is closer to FO, while ITS phylogenetic tree support FV is closer to FO species. EF-1a, beta-tubulin, RPB2 and CYP51C phylogenetic trees show that FOs from the same forma specialis are more likely to cluster together. For example, four FO species (phw815, phw808, Fo.5176 and Fo.cabbage), whose host is cabbage, are all clustered together in the EF-1a phylogenetic tree, while the ITS phylogenetic tree cannot display the closer relationship among the four FO species (Figure 2). It was noted that, with the exception of the ITS tree, all phylogenetic trees support FOP clustered together with Fo.cotton (the host is cotton). The hosts of FOP and Fo.hdv247 are cowpea and pea that belong to leguminous plants, but FOP and Fo.hdv247 did not cluster together in all phylogenetic trees, which reflects the complex relationship among FO species to some extent.

### 2.3. FOP Genome Assembly, Annotation and Comparative Genome Analysis

**Figure 3:**
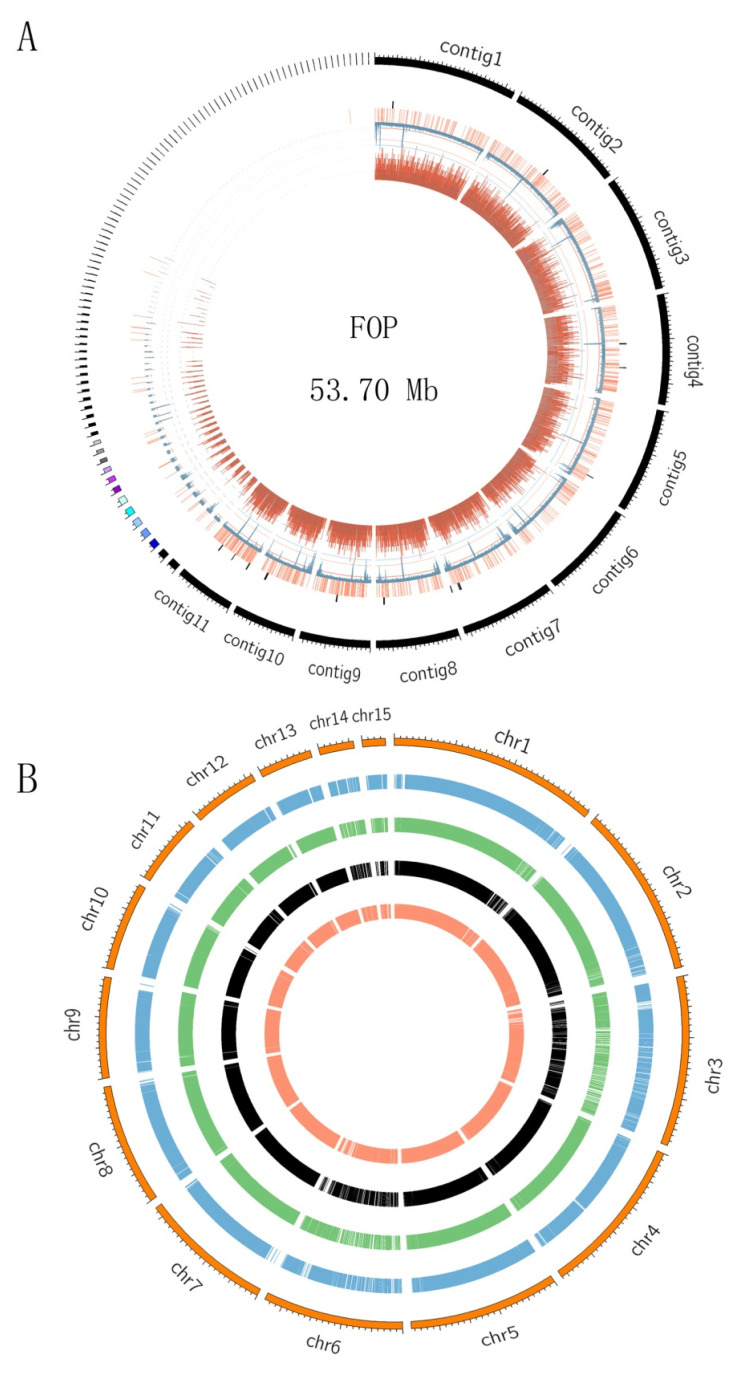
Genome features of FOP and comparative analysis among five FO species genomes. (**A**) Genome feature of FOP. The outermost circle is the contigs. The bar charts from outside to inside in turn are secondary metabolite gene clusters (black), secreted proteins (orange), density of repetitive sequence (blue) and gene density (dark red). (**B**) Comparative genome analysis of five FO genomes. The 15 chromosomes of FOL were used as references. The number out of the circle represents the chromosome number. The bar charts from outside to inside in turn are FOL (orange), FOCA (blue), FOCU (green), FOS (black) and FOP (light salmon).

The genome of FOP was sequenced using both Nanopore and Illumina technologies. We obtained 9.51 Gb (~158×) of long reads from the Nanopore platform, and 6.2 Gb (~103×) pair-end reads from the Illumina platform for FOP. The reads were assembled into 106 contigs, and the genome size of FOP was 53.70 Mb, with an N50 of 4.32 Mb (Figure 3A). To assess the assembly accuracy, we remapped the paired-end reads to the assembled FOP genome. The coverage rate for the FOP genome is 96.34%, indicating that FOP assembly covered almost all the genomic region. A total of 14,694 protein-coding genes were predicted for FOP. Among the predicted genes, 12,197 (83.01%) genes were functionally annotated. BUSCO assessment of the FOP genome showed that 1357 (94.31%) of the gene models were complete (Table 1), suggesting that the assemblies included most of the FOP genes.

We identified a total of 14 secondary metabolism gene clusters in the FOP genomes, including 8 NRPS gene clusters and 3 terpene gene clusters (Figure S3). Comparing the secondary metabolism gene clusters of FOP and FOL, we found that seven NRPS gene clusters showed syntenic relationship between FOP and FOL, indicating that NRPS gene clusters were conserved between the two FO genomes (Figure S4).

We used five FO genomes for comparative analysis, including FOCA (*Fusarium oxysporum* f. sp. *cabbage*, accession: GCA_014154955.1), FOL (*Fusarium oxysporum* f. sp. *lycopersicum,* accession: AAXH00000000), FOCU (the host is cucumber, accession: GCA_001702695.2), FOS (the host is strawberry, accession: GCA_016166325.2) and FOP in which the genomes of FOCA and FOL were chromosome-level genomes. Using FOL as the reference genome, we mapped the other four FO genomes (as query genomes) to fifteen FOL chromosomes. As shown in Table S1 and Figure 3B, the similar results can be seen in all four FO query genomes. The average sequence similarities of four chromosomes (chr3, chr6, chr14 and chr15 of FOL) were significantly lower than those of other chromosomes, with chr3 at 57.06%, chr6 at 53.65%, chr14 at 51.8% and chr15 at 58.92% (Table S1). The average gene density of the four chromosomes (2.18 genes/10 kb) was significantly lower than that of whole genome (3.02 genes/10 kb), while the average TE density of the four chromosomes (15.01/100 kb) was much higher than that of the overall average (4.89/100 kb) (Table S1). Ma et al. (2010) classify these four chromosomes as accessory chromosomes, in which chr14 may be associated with FO pathogenicity and forma specialis [21]. The sequence similarity of chr14 is 74.85% in FOCA, 55.63% in FOCU, 34.01% in FOS and 42.72% in FOP, indicating that chr14 is highly variable among species.

### 2.4. Comparative Analyses of Effectors among Five FO Genomes

**Figure 4:**
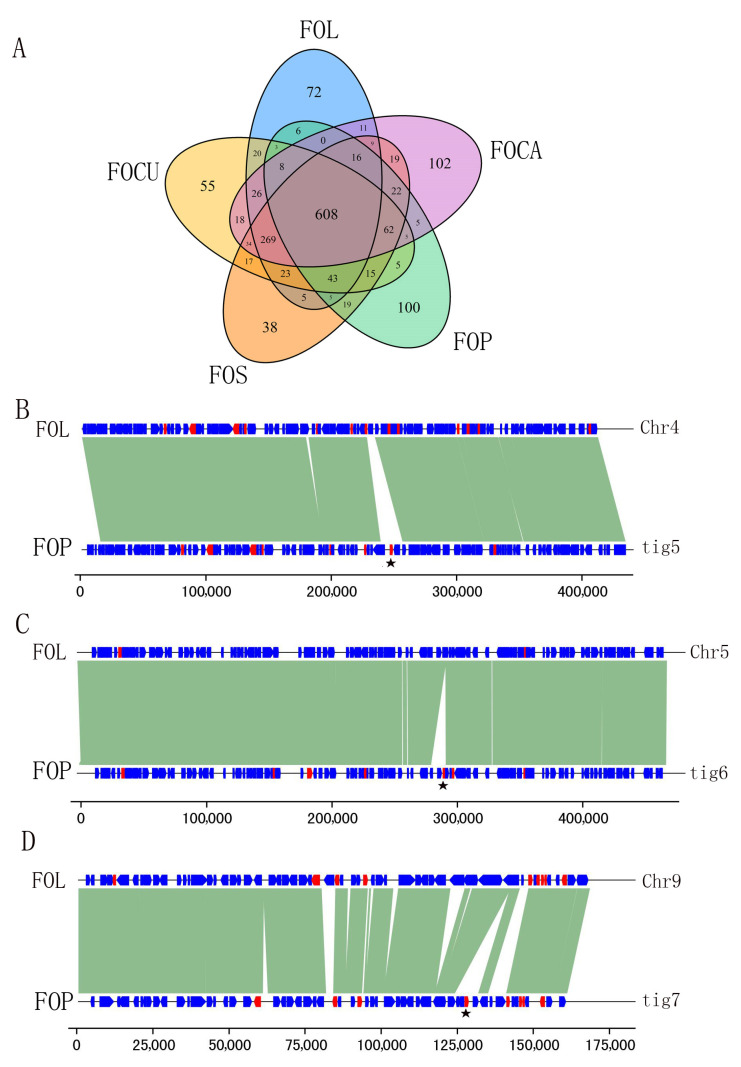
Predicted effector profiles in five FO species and comparative genomic analysis of FOP-specific effectors. (**A**) Venn diagram showing unique and shared effectors among five FO genomes. (**B**–**D**) Selected FOP-specific effectors associated with INDEL events. The black line above represents the chromosome number of FOL, and the one below represents the contig number of FOP. Blue arrows indicate genes and red arrows indicate effectors. The lines with the dark sea green color represent alignment between FOP and FOL genomes with high similarity (>80%). Black pentagrams indicate FOP-specific effectors. The bottom ruler is the length of the alignment sequences.

Effectors play important roles in FO pathogenicity. In this study, we identified 1348 putative effectors in the FOCA genome, 1296 in FOCU, 1286 in FOS, 1235 in FOL and 971 in FOP. Among these effectors, a total of 608 effectors were shared by 5 FO species (Figure 4A). By comparative genomic analysis among the five FO genomes, we identified 102 FOCA-specific, 55 FOCU-specific, 38 FOS-specific, 72 FOL-specific and 100 FOP-specific putative effectors (Table S2). For the FOL-specific putative effectors, 33 out of 72 were located in 4 accessory chromosomes, and 28 effectors were located on the terminal of core chromosomes (Table S3). When mapping the putative effectors of the other four FO species to the FOL genome, we also found that few effectors can be mapped to the accessory chromosomes in FOL. Our results showed that there were few FO conserved effectors on accessory chromosomes, and accessory chromosomes along with the terminal of core chromosomes were the main genome region where FO-specific effectors were produced.

By comparing the genomic regions of specific effectors, we found that large insertion or deletion (INDELs) events are one of the reasons for the emergence of these species-specific effectors. In 100 FOP-specific effectors, we identified 13 effectors associated with genome INDEL events (Table S2). As shown in Figure 4B, one INDEL of 17.01 kb in contig5 (region from 2,735,732 bp to 2,752,736 bp) of FOP resulted in the insertion of FOP-specific effector *EVM006761* compared with chromosome four in FOL. An insertion of 12.26 kb of contig6 was the source of FOP-specific effector *EVM008070* (Figure 4C). A 10.64 kb insertion in contig7 resulted in FOP-specific effector *EVM009981*. Our results indicate that INDEL events play an important role in the formation of species-specific effectors in FO. As a result, INDEL events may be associated with FO pathogenicity and forma specialis.

## 3. Discussion

In the control of fusarium wilt, accurate identification of FO forma specialis is essential for disease control [31]. Cowpea is an important vegetable in China. However, its production sustains heavy losses from fusarium wilt caused by FOP. The traditional methods of identifying FO are combined morphology analysis with ITS or EF-1a sequence similarity. The translation EF-1a and ITS sequences were used in these studies and many reports showed that compared with the ITS sequence, the EF-1a was a suitable genetic marker to distinguish between species. However, some studies have showed that EF-1a cannot fully reflect the genetic distance of FO [32,33,34]. Hannah et al. detected genetic diversity among 86 diverse FO isolates and found that the population based on the EF-1a genotype is not reflective of FO isolates’ genetic relatedness [35]. In this study, besides the ITS and EF-1a sequences, we used beta-tubulin, RPB2 and CYP51C sequences to analyze evolution relationship among FO species. Compared with the ITS sequence, EF-1a, beta-tubulin, RPB2 and CYP51C showed some similar results. For example, except for ITS trees, all phylogenetic tree supported FOP has the closest genetic relationship with Fo.cotton. Some FO species (the host is cabbage) of the same forma specialis were also clustered together in EF-1a, beta-tubulin, RPB2 and CYP51C phylogenetic trees. Therefore, these phylogenetic trees can reflect the genetic distance of FO species to a certain extent. Our results suggest that more conserved sequences should be used to analyze the genetic distance of FO species to obtain more accurate results. In this study, we combined morphology analysis, ITS and EF-1a sequence similarity with PCR to identify FOP, but only limited to a fusarium at genus level. At present, there are seven physiological races of FOP reported in the withering of *Phaseolus vulgaris* [36], and the different physiological races are mainly related to the geographical region. Based on the FOP genome sequence, we could develop an FOP-specific PCR primer to identify FOP at species level, and further develop specific primers for identifying physiological races of FOP in the future. 

When we compare the FOP genome to the FOL genome, the four ACs in the FOL genome are obvious. The sequence similarity of the four ACs is much less than that of the CCs. The sequence similarity of these four chromosomes is low, indicating that these four chromosomes may be related to their specialization and pathogenicity. It is noted that besides sequence similarity, sequence rearrangement is also a major reason for species evolution [17,28]. Sequence rearrangement leads to gene rearrangement, which may lead to products of new functions [21,23]. How the rearrangement of AC sequences affects the function of genes is still limited in FO. In particular, the number and order of effectors above the AC may affect the pathogenicity of pathogens. More research should be devoted to this study.

The products of secondary metabolic gene clusters (SMGCs) are essential for FO infection [37]. So far, most SMGCs in FO were annotated by genome-wide bioinformatics prediction, but their products have not been determined [38,39]. In this study, we identified 14 and 17 SMGCs in FOP and FOL, respectively, through bioinformatic analysis. Using comparative genomic analysis, we further found that most NRPS gene clusters are conserved in two genomes, indicating that the functions of these NRPS gene clusters are conserved in FO species. Most of the conserved gene clusters were located on the core chromosomes. We also found some SMGCs located on accessory chromosomes in the FOL genome. More studies will be required to find whether these SMGCs are related to the pathogenicity of FO.

Effector genes encode small in planta secreted proteins that are proposed to manipulate the host to promote colonization. Specifically, the non-autonomous DNA transposon miniature impala is associated with promoters of effector genes in FOL [28]. The number of effectors in different FO genomes varies, from less than 800 to over 2000. Ma et al. compared the effector numbers in different FOs and found that copy number variation of effector can explain the different number of effectors [21]. However, the reason for loss or gain of effector remains unclear. By comparing the effector loci between FOP and FOL, we found that large INDELs were the main reason for loss or gain of effectors between FOP and FOL. The loss or gain of effectors by large INDELs was detected in both FO genomes, which indicated that the effector gene loci in clusters have evolved relatively quickly.

## 4. Materials and Methods

### 4.1. The Morphology and Pathogenicity Test for the FOP Strain

The FOP strains were kindly provided by Xu Rongfeng from the culture collection center of the Fujian Agriculture and Forestry University. To confirm whether the strain was FOP, the morphology characteristics of the colonies, hyphae and microconidia were performed according to Liu et al. [40]. From the inclined tube in which the pathogen was stored, some mycelia were selected using an inoculation needle, transferred to a PDA medium containing three antibiotics (three antibiotics: streptomycin, chloramphenicol and lactic acid) and cultured in an incubator at 25 °C in the dark for 5–7 days. Aerial hyphae were white and flocculent, and the medium was pale purple in the middle and purple on the back. Two to three pathogen blocks were taken and put into a PL liquid medium containing three antibiotics. A large number of small conidia, which were oval under the microscope, were obtained by shaking at 180 rpm for 72 h at 25 °C.

The root-dipping method was used in the pathogenicity test according to lv et al [30]. The cowpea cultivar Fengjiang1, widely cultivated in southern China, was used for the FOP pathogenicity test. The cowpea seedlings were cultivated in a greenhouse at a temperature of 28 °C in the day and 20 °C at night until the two-leaf stage. The roots of the seedlings were dipped in the conidial suspension for 15 min, and then they were planted in plastic pots with a sterilized substrate and maintained in the greenhouse with a day temperature of 26–30 °C and a night temperature of 22–25 °C. Disease symptoms were measured 10 days after inoculation according to lv et al. [30] and three replicates were performed in the test for each FOP isolate test.

### 4.2. The Molecular Identification and Construction of Phylogenetic Trees for FOP

Molecular analysis was performed to include the FO-specific fragment and the ITS sequence. The primers for the FO-specific fragment were 5′-TCAATGATAGTGACAAGGGTTT and 5′-AATTTGCTGTGATAGGTGGAT, which can amplify a FO-specific DNA fragment with a size of 729 bp. The primers for the ITS sequence were universal 5’-TCCGTAGGTGAACCTGCGG (ITS1) and 5’-TCCTCCGCTTATTGATATGC (ITS4) PCR primers. The phylogenetic trees were constructed by using the ITS region, EF-1a gene, beta-tubulin gene, RPB2(RNA polymerase 2) gene and CYP51C sequences. The primers for the EF-1a gene were 5′-AACATGATCACTGGTAAT and 5′-TAAGCAGAAGCCCTTCGC. The primers for beta-tubulin were 5’-ATGCGTGAGATTGTAAGTACCTCT and 5’-GCACCGGACTGGCCGAAAACGAAGT. The primers for RPB2 were 5’-AGGTGTTGAACAGATATACCT and 5’-GTCATAGGACATACATACCT. The primers for CYP51C were 5’-CTACATCCCCAAATTCGTC and 5’-TTGGGATCAGGCTGTTCAAT.

All the PCRs in this study were carried out using 20 µL reaction mixtures, which consisted of 1 µL of genomic DNA (100 ng/µL), 2 µL of 10 × PCR Buffer, 0.5 µL of dNTPs (10 mmol), 0.5 µL of EasTaq polymerase (5 U/µL), 1.0 µL of each primer (10 μmol/L) of corresponding primer sets and the addition of PCR-grade water to final volume. The parameters for PCR were denaturing at 95 °C for 5 min, followed by 35 cycles of denaturing at 94 °C for 30 s, annealing at 52–59 °C for 30 s, polymerizing at 72 °C for 0.5–1.5 min and with a final extension at 72 °C for 10 min.

The nucleotide sequences of the ITS region, EF-1a gene, beta-tubulin gene, RPB2 gene and CYP51C sequences were aligned with ClustalW with default parameters. The phylogenetic tree was generated by the neighbor-joining (NJ) method with default parameters from the alignment of the nucleotide sequences with MEGA10. Bootstrap analysis with 1000 replications was performed to assess group support.

### 4.3. Genome Assembly and Annotation

The Nanopore original sequence was assembled with the Canu (v1.6) [41] assembler and DBG2LOC [42] after quality control and filtering of low-quality sequences. To improve the assembly result, Quickmerge software [43] was used to merge the two genome assemblies. The genome was first polished using Quiver followed by three rounds using Pilon [44]. 

Gene structure, function annotation and repeat annotation were carried out according to the operation manuals [45]. A strategy combining ab initio gene prediction and homology-based gene prediction was used for gene annotation. The repetitive sequences were annotated by combining the ab initio and homology-based methods. First, an ab initio repeat library was predicted for each genome with RepeatModeler. Second, this library was combined with Repbase (https://www.girinst.org/repbase, accessed on 25 August 2022) to identify all homologous repeats throughout the genome by RepeatMasker (https://www.repeatmasker.org, accessed on 25 August 2022) with WU-BLASTX as the search engine. We trained Augustus and SNAP using the high confident gene models from the results of PASA assembly, and GeneMark-ES was self-trained on the repeat-masked genome sequences. Homologous protein sequences from FO were downloaded from Broad institute (https://www.broadinstitute.org, accessed on 30 August 2022), and all of these sequences were mapped to each assembly with tBLASTN with an E-value cutoff of 1 × 10^−5^. Genewise (parameter: -gff -quiet -silent -sum) was used to refine the alignment. All results were integrated into consensus gene models using EvidenceModeler.

### 4.4. Effector Annotation and Comparative Genome Analysis

Potentially secreted proteins were identified using SignalP [46] after removing trans-membrane proteins based with TMHMM [47] and TargetP [48]. Species-specific effectors were identified by OrthoMcl software (v2.0.9). A whole-genome comparison was performed using the nucmer module of the MUMmer package (version 3.23) with the parameters –maxmatch [49]. The output of nucmer was filtered by delta-filtering (with the parameters -i 85-L 1000-1-r-q) to identify the one-to-one syntenic blocks between two genomes, and then a custom Perl script was used to identify the structural variations (INDEL events).

## 5. Conclusions

In this study, we provided the genome data of FOP and performed comparative genome analysis. Our results revealed that four accessory chromosomes in five FO showed low sequence similarity, low gene density and high repeat sequences content, indicating that variable accessory chromosomes are the main source of FO evolution. We identified a total of 100 FOP-specific effectors in FOP genomes and found 14 FOP-specific effector genes located in INDEL regions, suggesting that INDEL events can cause the emergence of species-specific effectors in FO. Our work contributed to our knowledge of FOP genome resources and can provide useful information for genome and effector evolution in FO species.

## Figures and Tables

**Table 1:** Statistics of the FOP assembly.

Statistics of Genome Assembly	FOP
length of genome assembly (Mb)	53.70
number of contigs	106
N50 of contigs (Mb)	4.32
total length of retrotransposons (Mb)	28.14
number of annotated genes	14,694
average gen length (bp)	1547
average CDS length (bp)	1203
average protein length (bp)	401
genome completeness (BUSCO)	94.31%

## Data Availability

FOP genome data can be downloaded from the Genome Warehouse in National Genomics Data Center (https://ngdc.cncb.ac.cn/?lang=en, accessed on 27 October 2022); Beijing Institute of Genomics (China National Center for Bioinformation) and Chinese Academy of Sciences (https://bigd.big.ac.cn/gwh, accessed on 27 October 2022) under accession number GWHBOXY00000000.

## Supplementary Materials

The following supporting information can be downloaded at: https://www.mdpi.com/article/10.3390/ijms24020963/s1.
