# Supplementary material for: The Genome of Fusarium oxysporum f. sp. phaseoli Provides Insight into the Evolution of Genomes and Effectors of Fusarium oxysporum Species
Source: Int J Mol Sci. 2023 Jan 4;24(2):963. doi: 10.3390/ijms24020963 (PMC9861946; doi:10.3390/ijms24020963)
Supplement: Supplementary file 1 [file ijms-24-00963-s001.zip › ijms-2032405-supplementary.pdf]

Table S1 statistics of sequence similarity, gene density and TE density

	FOCA	FOCU	FOS	FOP	average	Genedenstiy#1	TEdensity#2
chr1	91.23	91.87	87.73	89.76	90.14	3.24	2.76
chr2	88.25	87.95	87.43	88.43	88.01	3.25	1.34
chr3	68.3	49.95	52.8	57.22	57.06	2.22	8.43
chr4	90.7	94.47	92.02	91.92	92.27	3.25	0.69
chr5	93.13	95.17	94.68	94.36	94.33	3.06	0.65
chr6	65.26	51.59	47.26	50.49	53.65	2.19	8.17
chr7	88.26	95.88	94.57	95.27	93.49	3.15	0.61
chr8	91.01	94.53	94.63	94.47	93.66	3.23	0.58
chr9	85.37	95.51	92.32	92.48	91.42	3.15	1.12
chr10	87.87	93.22	88.6	89.16	89.71	3.21	0.90
chr11	88.46	94.86	91.46	91.28	91.51	3.7	1.45
chr12	87.83	89.72	90.19	89.31	89.26	3.63	1.21
chr13	88.41	94.28	91.04	91.17	91.22	3.63	2.01
chr14	74.85	55.63	34	42.72	51.8	2.08	27.52
chr15	72.7	55.77	47.47	59.74	58.92	2.24	15.93

#1: the sliding window is 10 kb

#2: the sliding window is 100 kb

Table S2 statistic of FOP-specific effectors

Gene id	Contigs	begin	end	orientation	FOP-specific
EVM000485	contig1	1785868	1786524	+	
EVM000675	contig1	2442284	2443398	+	
EVM000793	contig1	2809328	2811602	-	
EVM000915	contig1	3226819	3227468	+	
EVM001147	contig1	3982678	3983052	-	
EVM001160	contig1	4041349	4042462	-	
EVM001171	contig1	4085943	4086640	+	
EVM001254	contig1	4346816	4347717	-	
EVM001286	contig1	4443274	4444227	+	
EVM001294	contig1	4468295	4470935	+	√
EVM001440	contig1	5017794	5019124	+	
EVM001554	contig1	5379323	5379800	-	

EVM001721	contig1	5883741	5885649	-	
EVM001971	contig2	303442	305094	+	
EVM001976	contig2	323294	323822	+	
EVM002057	contig2	585065	586623	-	
EVM002582	contig2	2371423	2371913	-	
EVM002631	contig2	2527515	2527971	-	
EVM002882	contig2	3422350	3422743	+	
EVM002988	contig2	3749050	3750212	-	
EVM003126	contig2	4166137	4166463	-	
EVM003283	contig2	4645854	4646240	-	
EVM003508	contig3	569439	569783	-	
EVM003908	contig3	1875679	1876479	+	
EVM004235	contig3	3075753	3078708	+	
EVM004256	contig3	3137859	3138742	-	
EVM004263	contig3	3154133	3154813	-	
EVM004368	contig3	3490876	3491750	-	
EVM004405	contig3	3598741	3599288	-	
EVM004571	contig3	4180986	4183831	-	
EVM004666	contig3	4534937	4535458	+	
EVM004682	contig3	4603154	4605667	+	
EVM004901	contig4	709521	709905	+	
EVM005349	contig4	2216148	2216666	-	
EVM005434	contig4	2484396	2487033	-	√
EVM005826	contig4	3934330	3935236	+	
EVM005834	contig4	3954422	3954763	-	
EVM006252	contig5	1075656	1076001	+	
EVM006412	contig5	1565501	1566423	+	
EVM006460	contig5	1735056	1736297	-	
EVM006626	contig5	2274125	2274864	-	
EVM006674	contig5	2423195	2423917	+	
EVM006761	contig5	2743319	2744746	+	√
EVM006845	contig5	3000354	3001891	-	
EVM007017	contig5	3546865	3548199	+	
EVM007392	contig5	4747862	4748203	-	
EVM007639	contig6	376715	379369	+	
EVM007927	contig6	1284484	1285470	+	
EVM007973	contig6	1404639	1406177	+	
EVM008033	contig6	1625017	1626196	+	
EVM008038	contig6	1652427	1655475	+	
EVM008050	contig6	1697665	1698607	-	
EVM008070	contig6	1760491	1762003	+	√
EVM008073	contig6	1767975	1768697	+	
EVM008315	contig6	2634718	2636809	+	
EVM008502	contig6	3326381	3326587	+	

EVM008800	contig6	4363141	4365662	+	
EVM009331	contig7	927742	928102	-	√
EVM009404	contig7	1144505	1144987	-	
EVM009455	contig7	1329990	1330237	+	
EVM009698	contig7	2160017	2163595	-	
EVM009712	contig7	2216330	2217388	+	
EVM009743	contig7	2316598	2317691	+	
EVM009863	contig7	2727796	2728247	-	
EVM009883	contig7	2819733	2822273	+	
EVM009981	contig7	3127024	3127933	+	√
EVM010032	contig7	3306177	3306915	+	
EVM010084	contig7	3553250	3554849	-	√
EVM010109	contig7	3692039	3692284	-	√
EVM010134	contig8	47654	47929	+	√
EVM010335	contig8	1018515	1020347	-	√
EVM010579	contig8	1949683	1950447	+	
EVM010856	contig8	2898738	2900858	-	
EVM010924	contig8	3142393	3145102	-	
EVM010961	contig8	3264418	3265076	-	
EVM011188	contig8	3966608	3968464	-	√
EVM011446	contig9	897973	898809	+	
EVM011509	contig9	1072212	1073518	-	
EVM011538	contig9	1198071	1203990	+	
EVM011715	contig9	1738261	1739506	-	
EVM012033	contig9	2712186	2713450	+	
EVM012093	contig9	2905727	2906796	-	
EVM012751	contig10	2017631	2018260	-	
EVM012810	contig10	2215215	2217049	-	
EVM012914	contig10	2602972	2606720	-	√
EVM013074	contig11	525024	527039	+	
EVM013320	contig11	1265155	1265535	-	
EVM013350	contig11	1340635	1341462	-	
EVM013636	contig11	2214310	2214775	+	
EVM013665	contig11	2310551	2311888	+	
EVM013812	contig12	284335	286231	+	
EVM013842	contig13	43917	44864	-	
EVM013889	contig13	241727	242624	+	
EVM014168	contig18	119172	119716	-	
EVM014342	contig22	107531	107927	-	
EVM014459	contig28	8704	10607	+	
EVM014478	contig28	100292	100827	-	
EVM014589	contig36	43300	43919	+	√
EVM014598	contig37	12773	13695	+	
EVM014674	contig101	3033	3456	-	

Table S3 distribution of FOP-specific effectors on FOL chromosomes

effector	chrosome	beEVMin	end	oriatation	Chromosomes#
EVM00030	chr1	123700	124317	+	CT
EVM00465	chr1	1562599	1567414	-	
EVM01466	chr1	4967715	4969183	+	
EVM01855	chr1	6191105	6191644	+	
EVM02043	chr2	133853	134494	-	CT
EVM02044	chr2	135960	137597	+	CT
EVM02096	chr2	305425	306244	-	CT
EVM02487	chr2	1513212	1514318	+	
EVM03672	chr3	654517	654840	+	AC
EVM03747	chr3	1041645	1042257	-	AC
EVM03803	chr3	1301349	1301961	-	AC
EVM04012	chr3	2227272	2229122	+	AC
EVM04016	chr3	2245117	2247220	-	AC
EVM04139	chr3	2734483	2736266	-	AC
EVM04202	chr3	3000744	3001301	-	AC
EVM04228	chr3	3130909	3131466	+	AC
EVM04291	chr3	3385692	3386181	+	AC
EVM04294	chr3	3395668	3397451	+	AC
EVM04416	chr3	3900767	3902870	+	AC
EVM04420	chr3	3918850	3920700	-	AC
EVM04700	chr3	5231043	5232026	-	AC
EVM05963	chr4	3499183	3499927	-	
EVM06392	chr4	4868677	4869645	+	CT
EVM06490	chr4	5207091	5209639	-	CT
EVM07851	chr5	4468933	4470940	+	CT
EVM07988	chr5	4877283	4878389	+	CT
EVM07990	chr5	4882998	4884775	+	CT
EVM08026	chr6	136965	141233	+	AC
EVM08166	chr6	739343	741174	-	AC
EVM08243	chr6	1083309	1084421	+	AC
EVM08262	chr6	1185655	1187758	+	AC
EVM08322	chr6	1461961	1462518	+	AC
EVM08383	chr6	1717253	1717742	+	AC
EVM08386	chr6	1727229	1729012	+	AC
EVM08505	chr6	2214456	2216559	+	AC
EVM08509	chr6	2232550	2234400	-	AC
EVM08844	chr6	3801188	3801511	-	AC
EVM08901	chr6	4081270	4082021	-	AC
EVM08980	chr6	4491663	4492898	-	AC
EVM08994	chr6	4555153	4555725	-	AC

EVM09000	chr6	4588410	4589645	+	AC
EVM09134	chr7	384856	387324	+	CT
EVM09172	chr7	490710	492631	+	CT
EVM09265	chr7	813206	814039	-	CT
EVM10404	chr8	104485	104775	-	CT
EVM10426	chr8	208700	209608	+	CT
EVM11359	chr8	3305726	3306859	+	CT
EVM11436	chr8	3551768	3554979	-	CT
EVM11509	chr8	3782165	3783460	-	CT
EVM11514	chr8	3800849	3802051	+	CT
EVM12295	chr9	2306243	2307886	+	
EVM12401	chr9	2681278	2681874	+	CT
EVM12446	chr9	2804213	2808257	-	CT
EVM12524	chr9	3015308	3016149	+	CT
EVM12575	chr9	3149554	3152034	-	CT
EVM13459	chr10	2597688	2599302	-	CT
EVM13528	chr10	2813168	2813692	+	CT
EVM13854	chr11	811887	813625	+	CT
EVM13913	chr11	971881	973603	-	CT
EVM14126	chr11	1547091	1547909	-	
EVM14415	chr12	4657	6131	-	CT
EVM14852	chr12	1152330	1153343	-	
EVM14867	chr12	1189131	1190342	+	
EVM14869	chr12	1196631	1197446	-	
EVM14898	chr12	1280924	1281943	-	
EVM15519	chr13	812015	812749	-	CT
EVM15874	chr14	39637	40008	-	AC
EVM16089	chr15	341944	342799	+	AC
EVM16090	chr15	344158	345257	-	AC
EVM16158	chr15	680642	681749	+	AC
EVM16172	chr15	717690	718545	+	AC
EVM16173	chr15	719904	721003	-	AC

CT is core chromosome terminal and AC is accessory chromosomes

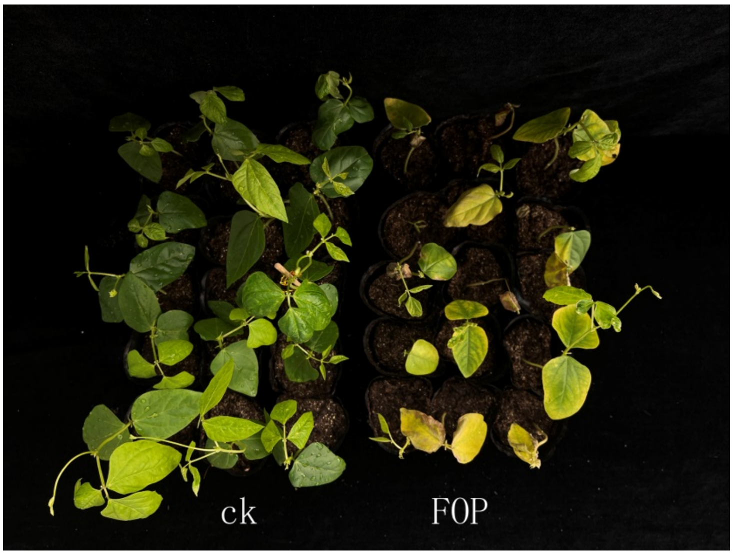

Figure S1 The pathogenicity test of FOP. The cowpea seedlings showed a typical fusarium wilt symptoms, including wilting, yellowing leaves and plant dwarfing, at 10 days after inoculated with FOP.

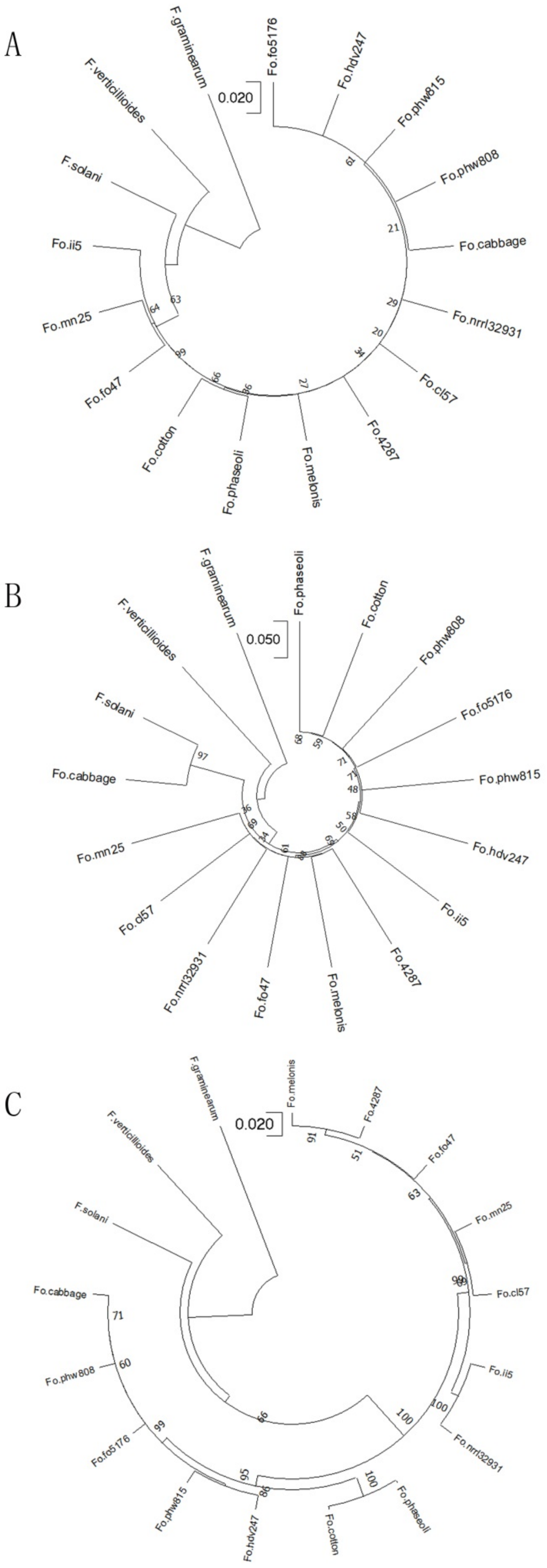

Figure S2. Phylogenetic tree constructed by beta-tubulin (A), RPB2 (B) and CYP51C (C) sequences using the neighbor-joining (NJ) method with bootstrap values shown at nodes. The names of the strains were listed in Figure 2.

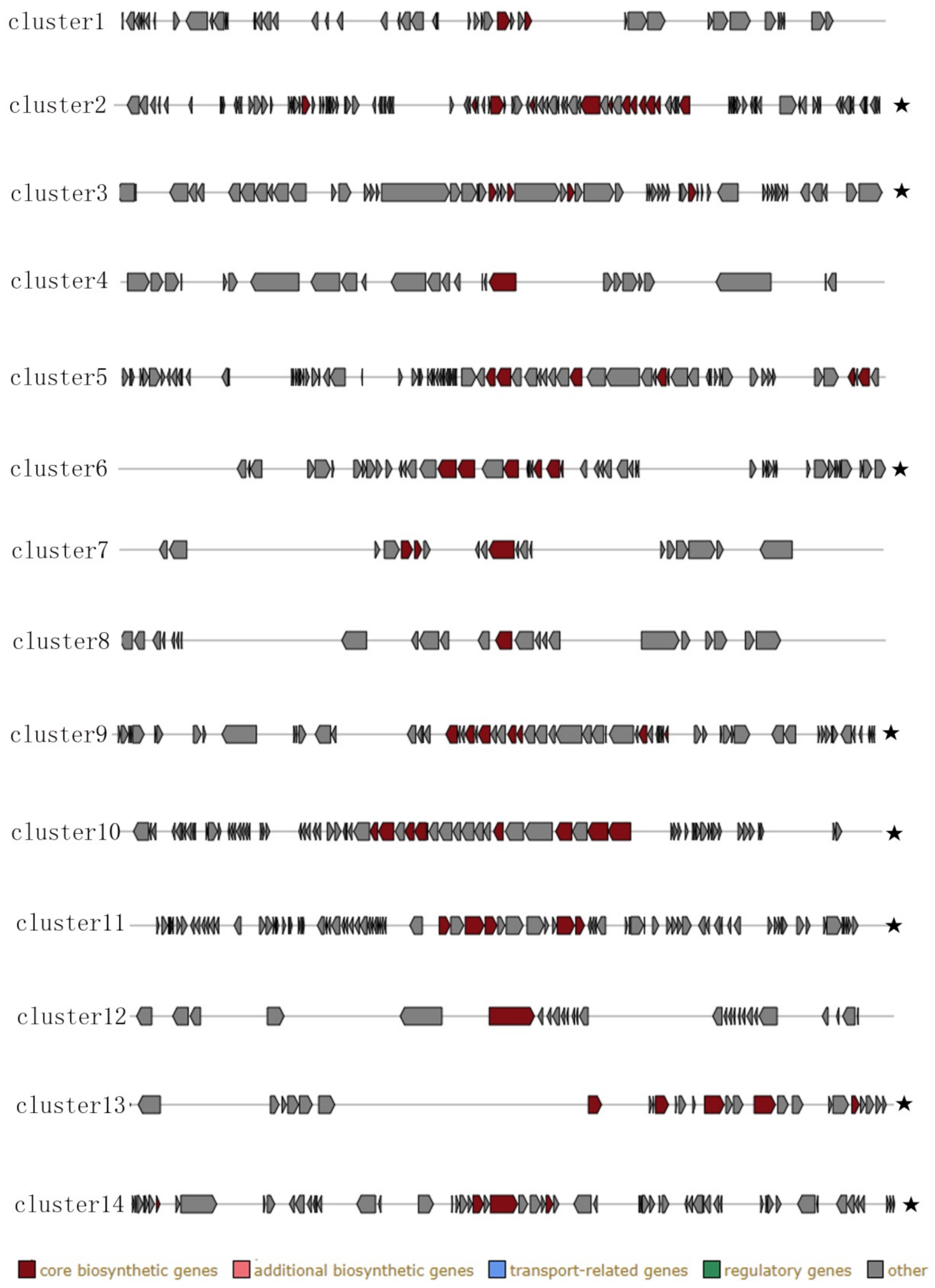

Figure S3 14 secondary metabolite clusters identified in FOP genome. The pentagrams represent NRPS gene clusters.

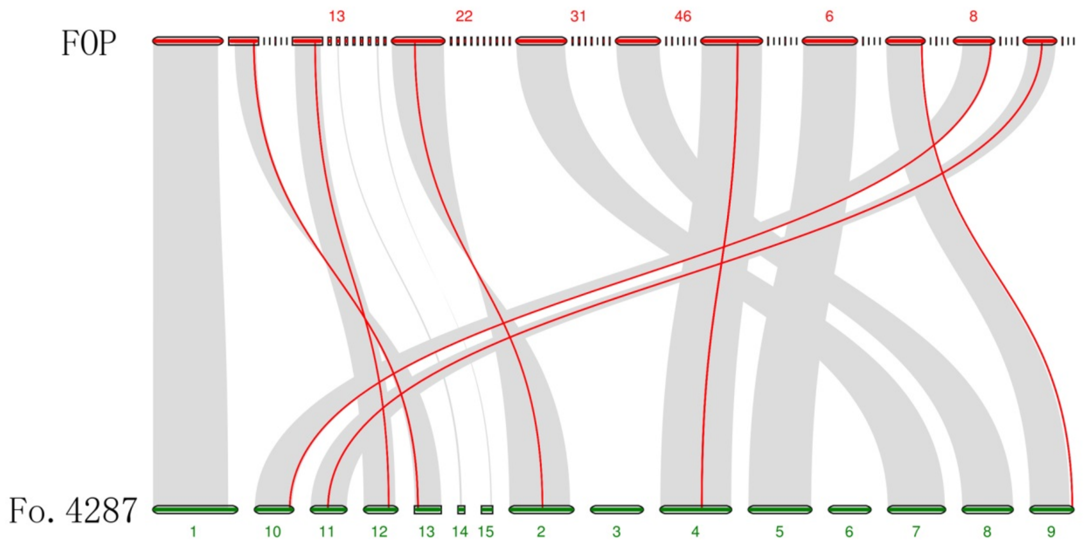

Figure S4. Synteny analysis of NRPS gene clusters between FOP and FOL(Fo.4287). Gray lines in the background indicate the collinear blocks between FOP and FOL genomes, and the red lines highlight the syntenic NRPS gene clusters. The red and green numbers represent contig nubmer (FOP) or chromosome number (FOL).
